# Physio-biochemical and morphological characters of halophyte legume shrub, *Acacia ampliceps* seedlings in response to salt stress under greenhouse

**DOI:** 10.3389/fpls.2015.00630

**Published:** 2015-08-31

**Authors:** Cattarin Theerawitaya, Rujira Tisarum, Thapanee Samphumphuang, Harminder P. Singh, Suriyan Cha-Um, Chalermpol Kirdmanee, Teruhiro Takabe

**Affiliations:** ^1^National Center for Genetic Engineering and Biotechnology, National Science and Technology Development Agency, Pathum Thani, Thailand; ^2^Department of Environment Studies, Panjab University, Chandigarh, India; ^3^Research Institute, Meijo University, Nagoya, Japan

**Keywords:** halophytic species, net photosynthetic rate, osmotic adjustment, Na^+^/K^+^ ratio, free proline, soluble sugar

## Abstract

*Acacia ampliceps* (salt wattle), a leguminous shrub, has been introduced in salt-affected areas in the northeast of Thailand for the remediation of saline soils. However, the defense mechanisms underlying salt tolerance *A. ampliceps* are unknown. We investigated various physio-biochemical and morphological attributes of *A. ampliceps* in response to varying levels of salt treatment (200–600 mM NaCl). Seedlings of *A. ampliceps* (25 ± 2 cm in plant height) raised from seeds were treated with 200 mM (mild stress), 400 and 600 mM (extreme stress) of salt treatment (NaCl) under greenhouse conditions. Na^+^ and Ca^2+^ contents in the leaf tissues increased significantly under salt treatment, whereas K^+^ content declined in salt-stressed plants. Free proline and soluble sugar contents in plants grown under extreme salt stress (600 mM NaCl) for 9 days significantly increased by 28.7 (53.33 μmol g^–1^ FW) and 3.2 (42.11 mg g^–1^ DW) folds, respectively over the control, thereby playing a major role as osmotic adjustment. Na^+^ enrichment in the phyllode tissues of salt-stressed seedlings positively related to total chlorophyll (TC) degradation (*R*^2^ = 0.72). Photosynthetic pigments and chlorophyll fluorescence in salt-stressed plants increased under mild salt stress (200 mM NaCl). However, these declined under high levels of salinity (400–600 mM NaCl), consequently resulting in a reduced net photosynthetic rate (*R*^2^ = 0.81) and plant dry weight (*R*^2^ = 0.91). The study concludes that *A. ampliceps* has an osmotic adjustment and Na^+^ compartmentation as effective salt defense mechanisms, and thus it could be an excellent species to grow in salt-affected soils.

## Introduction

Soil salinity is one of the major abiotic stresses that severely affect plant growth, development, and produce. It affects nearly 397 million hectares (Mha) of land world over, particularly in Asia-Pacific and Australia where around 195 Mha is salt-affected (Flowers et al., 1986; Rengasamy, 2006). Halophytic (salt-tolerant) species constituting only 1% of the world’s flora have evolved salt-defense mechanisms and show optimal growth in salt-affected soil (Flowers and Colmer, 2008). Such salt tolerance mechanisms include ion homeostasis system via salt glands/salt bladders, osmoregulation system such as free proline, glycinebetaine, mannitol and soluble sugars, hormonal regulation, and antioxidant machinery (Hasegawa et al., 2000; Flowers et al., 2010; Shabala, 2013; Shabala et al., 2014). Na^+^ homeostasis via antiporter proteins, i.e., Na^+^/H^+^ exchanger (NHXs) in halophytic species has been well established as a major channel to manage the Na^+^ influx from the soil solution into root cell and translocate via xylem loading to other organs, leading to increased Na^+^/K^+^ ratio (Flowers and Colmer, 2008; Li et al., 2008). Alternatively, free proline and total soluble sugar enrichments in halophytic plants have been reported as major osmolytes when plants are exposed to soil salinity (Yokota, 2003; Patel et al., 2010; Theerawitaya et al., 2014). In the case of physiological changes, photosynthetic pigment, i.e., chlorophyll a and b degrades depending on the degree of salt levels in the soil solution, especially in extreme salt stress (10 dS m^–1^) (Giri et al., 2003), causing to reduce net photosynthetic rate, especially in extreme salt stress (Takemura et al., 2000).

Halophytes have been regarded as potential new crops for use as forage, vegetable, and oilseed crop. However, the potential utilization of halophytic species to grow in salt-affected soil and to facilitate saline soil phytoremediation depends on several factors such as salt accumulation, relative growth rate and biomass conversion, multipurpose utilization, and economic returns to the farmers (Panta et al., 2014). *Acacia ampliceps* (salt wattle), a native of Australia, is a multipurpose plant species largely used for livestock feeding (based on 120–170 g kg^–1^ crude protein; Dynes and Schlink, 2002) and biomass production (23.34% dry biomass; Ashraf et al., 2012). In addition, it has the potential for saline soil remediation and grows well in the soil with EC_*e*_ ≥ 16 dS m^–1^ (Ansari et al., 2007). Previously, using multivariate cluster analyses of biomass productivity of *Acacia* species in salt-affected soil, *A. ampliceps* or salt wattle has been found to have the maximum biomass production (39.69 ton ha^–1^) than the other species: *A. karroo* (2.39 ton ha^–1^), *A. stenophylla* (0.53 ton ha^–1^), *A. seyal* (0.27 ton ha^–1^), and *A. asak* (0.18 ton ha^–1^) (Aref et al., 2003). In Thailand, *A. ampliceps*, a leguminous shrub, has been introduced in salt-affected areas in the northeast of Thailand for the remediation of saline soils (Wongpokkhom et al., 2008). However, the defense mechanisms underlying salt tolerance in *A. ampliceps* are largely unknown. However, no study has been conducted to investigate physiological and biochemical mechanisms underlying salt tolerance mechanisms in *A. ampliceps.* We, therefore, conducted a series of experiments to investigate the biochemical, physiological, and morphological attributes of *A. ampliceps* grown in response to extreme salt concentrations under greenhouse conditions.

## Materials and Methods

### Plant Material and Salt Treatments

Seeds of salt wattle (*Acacia ampliceps* Maslin) provided by Land Development Department, Ministry of Agriculture and Cooperatives, Thailand, were disinfected with 15% sodium hypochlorite for 30 min followed by washing three times with tap water and then with distilled water. Disinfected seeds were germinated in the plastic tray (30 × 60 × 5 cm) containing 1 kg of garden soil (sandy loam; EC = 2.687 dS m^–1^; pH = 5.5; organic matter = 10.36%; total nitrogen = 0.17%; total phosphorus = 0.07%; total potassium = 1.19%) under greenhouse conditions. 15-day old seedlings were transferred into plastic bag (W × L × H; 4 × 4 × 12 cm) containing garden soil (as above) and grown under controlled environmental conditions (80 ± 5% relative humidity, 500–1,000 μmol m^–2^ s^–1^ photosynthetic photon flux density, 10 ± 2 h d^–1^ photoperiod from sunlight and 28 ± 2°C ambient temperature) for 7 weeks. The experiment was conducted in the greenhouse of the Thailand Science Park, Pathum Thani, Thailand (Latitude 14°01′12″N Longitude 100°31′12″E).

Uniform-sized seedlings (25 ± 2 cm in plant height) were selected as initial material. Sodium chloride (NaCl) concentration in the soil solution was adjusted to 0 (control), 200 (mild stress), 400, and 600 mM (high stress). After 9 days, growth characters, Na^+^, K^+^, and Ca^2+^ contents, soluble sugar content, free proline content, photosynthetic pigments, chlorophyll fluorescence, net photosynthetic rate (P_*n*_), stomatal conductance (g_*s*_), and transpiration rate (E) in the phyllodes were measured (*n* = 6).

### Growth Performances

Shoot height (SH), root length (RL), number of phyllodes (NL), number of roots (NR), fresh weight (FW), dry weight (DW), and phyllode leaf area (LA) of seedlings were measured. Salt wattle seedlings were dried at 80°C in a hot-air oven for 2 days and then incubated in desiccator before the measurement of DW. Phyllode area was measured using a Root/Leaf Area Meter DT-scan (Delta-Scan Version 2.03, Delta-T Devices, Ltd, Cambridge, UK).

### Na^+^, K^+^, and Ca^2+^ Assay

Na^+^, K^+^, and Ca^2+^ were assayed following the modified method of Tanaka et al. (1999) and Hossain et al. (2006). In brief, phyllode tissues of salt-stressed seedlings were washed by deionized water to remove surface contaminating Na^+^. The tissues were ground into powder in liquid nitrogen, extracted with boiling distilled water, and centrifuged at 10,000 × g for 10 min. The supernatant was filtered through a 0.45 μm membrane filter (VertiPure^™^, Vertical^®^). Cellular Na^+^, K^+^, and Ca^2+^ concentrations were determined using HPLC (Waters Associates, Millford, MA, USA) coupled with 432 Conductivity Detector and WATER IC-PACK^™^ ion-exclusion column (Waters Associates, Millford, MA, USA). Nanopure water was used as mobile phase with 0.6 mL min^–1^ flow rate. Na^+^, K^+^, and Ca^2+^ (Sigma, USA) were used as standard.

### Total Soluble Sugars Determination

Total soluble sugars (sucrose, glucose, and fructose) in the second fully expanded phyllode (from shoot tip) were analyzed according to the modified method of Karkacier et al. (2003). In a pre-cooled mortar, 100 mg tissue was ground with liquid nitrogen, extracted with 1 mL of deionized water, vigorously shaken for 15 s, sonicated for 15 min and then centrifuged at 12,000 rpm for 15 min. The supernatant was filtered through a 0.45 μm membrane filter (VertiPure^™^, Vertical^®^) and stored at –20°C prior to the measurement of total soluble sugars content using HPLC. A volume of 40 μL of crude extracts was automatically injected into a Waters HPLC fitted with a Waters 600 pump using a MetaCarb 87°C column equipped with a guard column. Deionised water was used as the mobile phase at a flow rate of 0.5 mL min^–1^. The online detection was performed using a Waters 410 differential refractrometer detector, and the data was analyzed by Empower^®^ software. Sucrose, glucose, and fructose (Fluka, USA) were used as standards.

### Free Proline Assay

Free proline in the second fully expanded phyllode (from shoot tip) was extracted and analyzed according to the method of Bates et al. (1973). Fifty milligram of fresh material was ground with liquid nitrogen in a mortar. The homogenate powder was mixed with 1 mL of aqueous sulfosalicylic acid (3%, *w/v*), and the solution was filtered through filter paper (Whatman No 1, England). The extracted solution was reacted with an equal volume of glacial acetic acid and ninhydrin reagent (1.25 mg ninhydrin in 30 mL glacial acetic acid and 20 mL 6 M H_3_PO_4_) and incubated at 95°C for 1 h. The reaction was terminated by placing the container in an ice bath. The reaction mixture was mixed vigorously with 2 mL of toluene. After cooling to 25°C, the chromophore was measured at 520 nm by UV-VIS spectro-photometer (HACH DR/4000; Model 48000, HACH Company, Loveland, CO, USA) using L-proline as a calibration standard.

### Photosynthetic Pigments

Chlorophyll a (Chl_*a*_), chlorophyll b (Chl_*b*_), and TC contents in the second fully expanded phyllode were analyzed following the method of Shabala et al. (1998). 100 milligram of phyllode tissue was homogenized in glass vials using 10 mL of 95.5% acetone and blended using a homogenizer. The glass vials were sealed with parafilm^®^ to prevent evaporation and then stored at 4°C for 48 h. Chl_*a*_ and Chl_*b*_ concentrations were measured at 662 nm and 644 nm using UV-VIS spectrophotometer against acetone (95.5%) as a blank.

### Chlorophyll Fluorescence

Chlorophyll fluorescence emission was measured from the adaxial surface of the second fully expanded phyllode using a fluorescence monitoring system (FMS 2; Hansatech Instruments Ltd., Norfolk, UK) in the pulse amplitude modulation mode (Loggini et al., 1999). A phyllode, kept in dark for 30 min, was initially exposed to the modulated measuring beam of far-red light (LED source with typical peak at wavelength 735 nm). Original (F_0_) and maximum (F_*m*_) fluorescence yields were measured under weak modulated red light (<0.5 μmol m^–2^ s^–1^) with 1.6 s pulses of saturating light (>6.8 μmol m^–2^ s^–1^ PAR) and calculated using FMS software for Windows^®^. The variable fluorescence yield (F_*v*_) was calculated using the equation: F_*v*_ = F_*m*_–F_0_. The ratio of variable to maximum fluorescence (F_*v*_/F_*m*_) was calculated as the maximum quantum yield of PSII photochemistry. The photon yield of PSII (Φ_*PSII*_) in the light was calculated as: Φ_*PSII*_ = (F_*m*_′-F)/F_*m*_′ after 45 s of illumination, when steady state was achieved (Maxwell and Johnson, 2000).

### Net Photosynthetic Rate (P_*n*_), Stomatal Conductance (g_*s*_) and Transpiration Rate (E)

Net photosynthetic rate (P_*n*_; μmol m^–2^s^–1^), stomatal conductance (g_*s*_; μmol CO_2_ m^–2^ s^–1^), and transpiration rate (E; mmol m^–2^ s^–1^) in the second fully expanded phyllode were measured using a portable photosynthesis system fitted with an infra-red gas analyzer (Model LI 6400, LI-COR^®^ Inc., Lincoln, NE, USA). The g_*s*_ and E were measured continuously by monitoring the content of the air entering and existing in the IRGA headspace chamber, according to Cha-um et al. (2007). The sample chamber was set at 500 μmol s^–1^ air-flow rate and 25°C of chamber temperature. The light intensity was adjusted to 1,000 μmol m^–2^ s^–1^ PPFD of 6400-02B red-blue LED light source.

### Experiment Design and Statistical Analysis

The experiment was arranged as Completely Randomized Block Design (CRBD) with six replicates (*n* = 6). The mean values obtained were compared using Tukey’s HSD and analyzed by SPSS software.

## Results

### Morphological and Growth Performances

**FIGURE 1:**
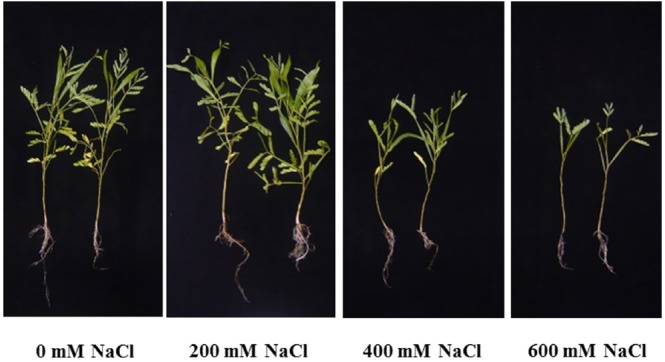
**Plant morphological characters of ***Acacia ampliceps*** seedlings grown under salt stress conditions for 9 days**.

**FIGURE 2:**
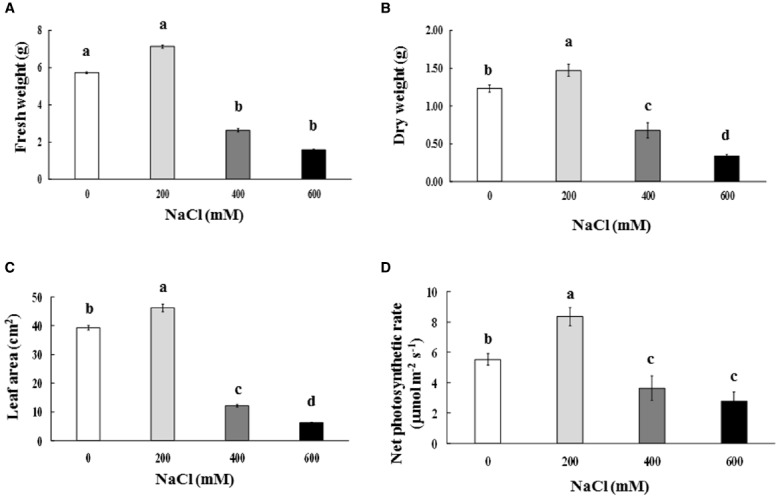
**Fresh weight (A), dry weight (B), phyllode leaf area (C), and net photosynthetic rate (D) of ***Acacia ampliceps*** seedlings grown under salt stress conditions for 9 days.** Data presented as mean ± SE. Different letters represent significant difference at *p* ≤ 0.01 according to Tukey’s HSD test.

Leaf color of control (0 mM NaCl) and mild salt-stressed plants (200 mM NaCl) was green. In contrast, phyllodes and the bipinnate leaves (true older leaves) turned yellow in the plants grown under extreme salt stress (400–600 mM NaCl) for 9 days. The overall growth characters declined in plants subjected to extreme salt stress for 9 days (Figure 1). SH, RL, NL, NR, FW, DW, and phyllode LA of salt wattle grown under 200 mM NaCl stress increased; however, these declined significantly when plants were subjected to 400–600 mM NaCl for 9 days (Table 1 and Figures 2A–C). SH, RL, NL, NR, shoot FW, and root FW in extreme salt-stressed (600 mM NaCl) plants were sharply dropped by 25, 23, 60, 45, 78, and 74%, respectively, compared to plants growing under mild stress (200 mM NaCl; Table 1). In addition, the salt-sensitive parameters such as NL, FW, and DW were decreased in plants exposed to 400 mM NaCl for 9 days, similar result to 600 mM. The growth and development of salt wattle plants under mild salt stress were enhanced and better than those under control condition (0 mM NaCl; Table 1).

**TABLE 1:** **Shoot height (SH), root length (RL), number of phyllodes (NL), number of roots (NR), shoot fresh weight (SHFW), and root fresh weight (RTFW) of ***Acacia ampliceps*** seedlings grown under salt stress conditions for 9 days**.

**NaCl (mM)**	**SH (cm)**	**RL (cm)**	**NL**	**NR**	**SHFW (g)**	**RTFW (mg)**
0	33.1 ± 1.87ab	14.1 ± 1.83ab	6.4 ± 0.60ab	20.0 ± 1.76ab	5.47 ± 0.12a	263 ± 3.4b
200	36.7 ± 1.58a	16.3 ± 1.42a	9.4 ± 1.12a	29.8 ± 1.66a	6.59 ± 0.21a	543 ± 3.9a
400	29.6 ± 0.77ab	13.3 ± 0.82ab	4.0 ± 0.32b	20.0 ± 2.11ab	2.45 ± 0.25b	185 ± 2.5c
600	27.5 ± 1.25b	12.5 ± 1.23b	3.8 ± 0.37b	16.4 ± 2.35b	1.45 ± 0.31b	139 ± 1.2c

Data presented as mean ± SE. Different letters in each column are significantly different at p ≤ 0.01 according to Tukey’s HSD test.

### Na^+^, K^+^, and Ca^2+^

**FIGURE 3:**
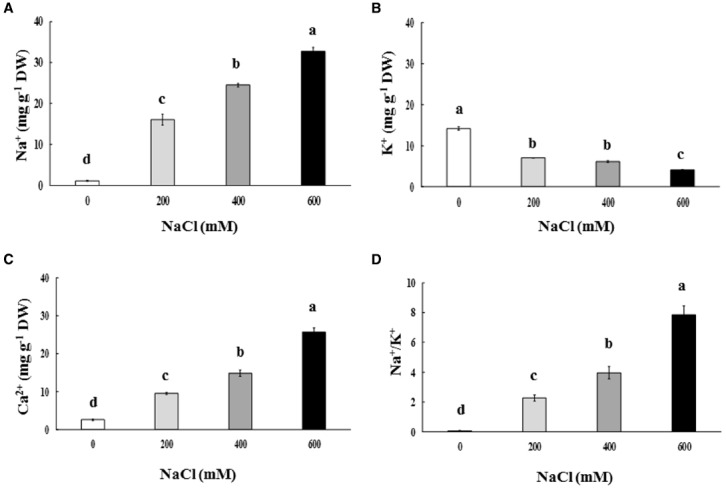
**Na^+^(A), K^+^(B), and Ca^2+^ content (C) and Na^+^/K^+^ ratio (D) of ***Acacia ampliceps*** seedlings grown under salt stress conditions for 9 days.** Data presented as mean ± SE. Different letters represent significant difference at *p* ≤ 0.01 according to Tukey’s HSD test.

Na^+^ and Ca^2+^ contents in the phyllode tissues of salt-stressed plants were continuously enriched, whereas K^+^ content declined significantly, depending on the NaCl concentration in the soil solution (Figures 3A–C). Na^+^/K^+^ in the phyllode leaves exhibited a trend similar to Na enrichment when plants were subjected to salt stress (Figure 3D). Under the extreme salt stress (600 mM NaCl), Na^+^ content in the phyllode tissues was peaked to ∼33 mg g^–1^ DW, thereby resulting in an increase in Na^+^/K^+^ ratio by 7.8 fold (Figure 3). In addition, Na^+^ and Ca^2+^ content in the phyllode tissues of mild salt-stressed plants (200 mM NaCl) was 16.1 and 9.5 mg g^–1^ DW, respectively.

### Free Proline and Total Soluble Sugar

**FIGURE 4:**
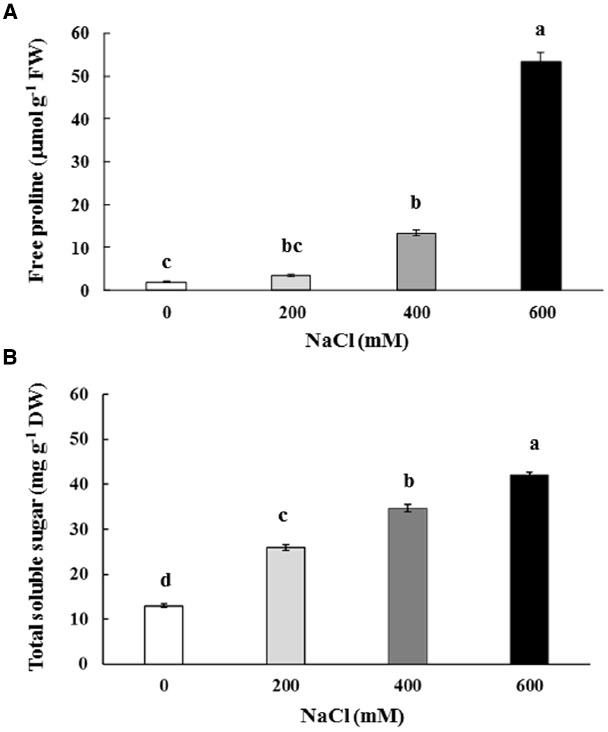
**Free proline (A) and total soluble sugar (B) content of ***Acacia ampliceps*** seedlings grown under salt stress conditions for 9 days.** Data presented as mean ± SE. Different letters represent significant difference at *p* ≤ 0.01 according to Tukey’s HSD test.

Free proline content in the phyllodes of salt-stressed plants was increased significantly, especially in plants under extreme stress. It increased by 7.1 (13.18 μmol g^–1^ FW) and 28.7 fold (53.33 μmol g^–1^ FW) in plants growing under 400 and 600 mM NaCl, over that in the control plants (1.86 μmol g^–1^ FW; Figure 4A). Similarly, the total soluble sugar were continuously accumulated in the phyllodes of salt-stressed plants and peaked to 42.1 mg g^–1^ DW (∼3.2 fold of control) in plants grown under 600 mM NaCl (Figure 4B). Sucrose, glucose, and fructose contents in salt-stressed plants were increased in relation to degree of salt stress. The sucrose, glucose, and fructose in plants grown under 600 mM NaCl were 2.2, 3.7, and 5.1 fold of control, respectively (Table 2). The accumulation of glucose and fructose was comparatively less in plants under 200 mM NaCl, where it increased by 2.3 and 3.6 fold, respectively, over that in the control plants.

**TABLE 2:** **Sucrose (Suc), glucose (Gluc), fructose (Fruc), stomatal conductance (g_***s***_), and transpiration rate (E) of ***Acacia ampliceps*** seedlings grown under salt stress conditions for 9 days**.

**NaCl (mM)**	**Suc (mg g^–1^ DW)**	**Gluc (mg g^–1^ DW)**	**Fruc (mg g^–1^ DW)**	**g_*s*_ (μmol H_2_O m^–2^ s^–1^)**	**E (mmol H_2_O m^–2^ s^–1^)**
0	6.61 ± 0.13c	3.94 ± 0.12c	2.55 ± 0.21c	5.51 ± 0.13b	1.63 ± 0.12b
200	7.79 ± 0.08bc	9.04 ± 0.41b	9.15 ± 0.30b	10.44 ± 0.56a	3.13 ± 0.14a
400	9.42 ± 0.15b	12.51 ± 0.59a	12.78 ± 0.20a	3.98 ± 0.13b	1.41 ± 0.18b
600	14.37 ± 0.62a	14.73 ± 0.79a	13.01 ± 0.23a	2.79 ± 0.12b	0.83 ± 0.08b

Data presented as mean ± SE. Different letters in each column are significantly different at p ≤ 0.01 according to Tukey’s HSD test.

### Photosynthetic Abilities

Chl_*a*_, Chl_*b*_, and TC in the phyllodes were enhanced in plants grown under mild salt stress (200 mM NaCl; Table 3). However, Chl_*a*_, Chl_*b*_, and TC were degraded by 48.3, 29.9, and 39.8% of control, respectively, under extreme salt stress (600 mM NaCl; Table 3). In addition, Chl_*a*_, a sensitive parameter, was significantly decreased when plants were exposed to 400 mM NaCl, whereas Chl_*b*_ was maintained. The maximum quantum yield of PSII (F_*v*_/F_*m*_) and photon yield of PSII (Φ_*PSII*_) declined when plants were subjected to extreme salt stress (400–600 mM NaCl) for 9 days. Under extreme salt stress, F_*v*_/F_*m*_ and Φ_*PSII*_ were diminished by 17.0 and 13.5%, respectively, compared to that in mild salt-stressed plants (Table 3). Net photosynthetic rate (P_*n*_) in mild salt-stressed plants was increased and then declined significantly at greater salt stress in a concentration-dependent manner (Figure 2D). Similarly, stomatal conductance (g_*s*_) and transpiration rate (E) in mild salt-stressed plants were peaked to 10.44 μmol H_2_O m^–2^ s^–1^ and 3.13 mmol H_2_O m^–2^ s^–1^, whereas these declined by 73.3 and 73.5%, respectively, when plants were exposed to 600 mM NaCl (Table 2).

**TABLE 3:** **Chlorophyll a (Chl_***a***_), chlorophyll b (Chl_***b***_), and total chlorophyll (TC) concentration, maximum quantum yield of PSII (F_***v***_/F_***m***_), and photon yield of PSII (Φ_***PSII***_) of ***Acacia ampliceps*** seedlings grown under salt stress conditions for 9 days**.

**NaCl (mM)**	**Chl_*a*_ (μg g^–1^ FW)**	**Chl_*b*_ (μg g^–1^ FW)**	**TC (μg g^–1^ FW)**	**F_*v*_/F_*m*_**	**Φ_*PSII*_**
0	59.15 ± 1.95b	70.05 ± 2.15ab	129.19 ± 4.43b	0.871 ± 0.005a	0.788 ± 0.012a
200	95.98 ± 6.63a	82.67 ± 3.87a	178.65 ± 5.56a	0.873 ± 0.003a	0.793 ± 0.012a
400	52.71 ± 0.50b	69.14 ± 1.90ab	121.85 ± 3.44b	0.788 ± 0.003b	0.715 ± 0.007b
600	49.64 ± 0.42b	57.99 ± 1.42b	107.62 ± 1.75b	0.725 ± 0.001c	0.686 ± 0.021c

Data presented as mean ± SE, Different letters in each column are significantly different at p ≤ 0.01 according to Tukey’s HSD test.

### Relationships Between Biochemical and Physiological Changes

**FIGURE 5:**
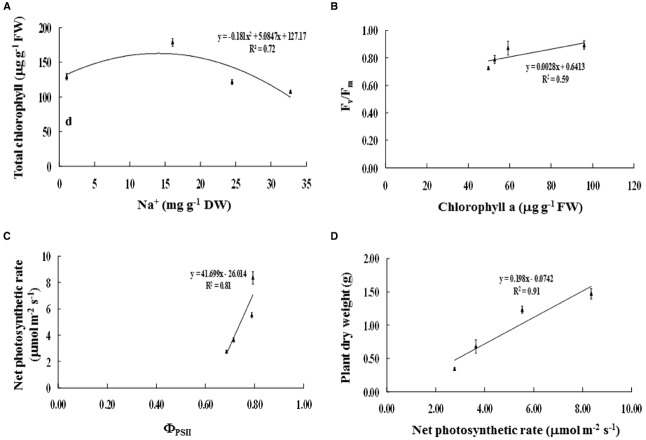
**Relationships between (A) Na^+^ and total chlorophyll content, (B) chlorophyll a content and maximum quantum yield of PSII (F_***v***_/F_***m***_), (C) photon yield of PSII (Φ_***PSII***_) and net photosynthetic rate (P_***n***_), and (D) net photosynthetic rate and plant dry weight of ***Acacia ampliceps*** seedlings grown under salt stress conditions for 9 days.** Error bars represent ± SE.

Na^+^ enrichment in the phyllode tissues of salt-stressed plants was positively related to TC degradation (*R*^2^ = 0.72; Figure 5A). Consequently, Chl_*a*_ degradation was positively correlated with reduction in maximum quantum yield of PSII (F_*v*_/F_*m*_) (*R*^2^ = 0.59; Figure 5B). Likewise, the diminishing of PSII photon yield (Φ_*PSII*_) was strongly related to decreased net photosynthetic rate (P_*n*_) (*R*^2^ = 0.81; Figure 5C), leading to declined plant DW (*R*^2^ = 0.91; Figure 5D).

## Discussion

The growth performance of *A. ampliceps* was better exhibited in the low-salt concentration; however, it declined depending on the degree of salt contamination in the soil. Previously, the survival percentage of *A. ampliceps* was reported to be the maximum (98.1%) with 127.2 cm SH, when grown in the saline soil habitat for 9 months (Shirazi et al., 2006). The mortality of *A. ampliceps* grown under hydroponic culture was only 7% (retaining 38% DW of control) when plants were exposed to 1.5% NaCl (257 mM) and increased to 47% (with only 22% DW of control) under 2.0% NaCl (342 mM) (Yokota, 2003). Our findings are corroborated by an earlier study reporting improved growth characters like, shoot FW, shoot DW, root FW, and root DW- of *A. ampliceps* under mild salt stress (EC_*e*_ = 10 dS m^–1^) in hydroponic culture and then declined under severe salt stress (EC_*e*_ = 20–30 dS m^–1^), especially at pH 9.5 (Azhar et al., 2013). In contrast, SH, RL, shoot FW, shoot DW, root FW, and root DW of *A. ampliceps* declined in plants exposed to 100–300 mM NaCl for 2 days (Abbas et al., 2013). Also, Marcar and Crawford (2011) observed a decline in SH in several provenances of *A. ampliceps* under 150 mol m^–3^ salt treatment and saline waterlogging conditions. Parallel to our findings, SH and stem diameter of *A. ampliceps* were increased in Riyadh field trials, leading to an increase in the final biomass yield at 39.69 ton ha^–1^ (Aref et al., 2003). In the salt-affected fields (EC_*e*_ = 13.9 dS m^–1^), SH and the number of branch per plants were enhanced, depending on the cultivation period (6–24 months; Abbas et al., 2015). The number of leaves is a good indicator to identify the mortality of plants grown under salt stress. Mahmood et al. (2009) suggested that the number of leaves of *A. ampliceps* were decreased by 10, 21, and 37% over the control, when subjected to EC_*e*_ 10, 20, and 30 dS m^–1^, respectively, for 2 years.

In our study, Na^+^ and Ca^2+^ in phyllodes of *A. ampliceps* were continuously enriched depending on the salt concentration in the soil solution, whereas K^+^ was declined, thereby resulting in increased Na^+^/K^+^ ratio. In halophytic species, Na^+^ was enriched in vacuole organelles, relating to activities of Na^+^/H^+^ antiporter (NHXs) and vacuolar H^+^-ATPase (VH^+^-ATPase) localization on vacuolar membrane (Barkla et al., 2002; Wang et al., 2015). These are in conformity with previous findings of Marcar et al. (1991) and Abbas et al. (2013) who reported increased Na:K or reduce K:Na ratio, respectively. Na^+^ content in new phyllode (young) of *A. ampliceps* grown under salt stress (EC_*e*_ = 40 dS m^–1^ in summer season) was peaked and greater than that in older phyllodes (Shirazi et al., 2001). A similar observation (Na^+^ accumulation and K^+^ reduction) has been demonstrated in other acacia species (*A. auriculiformis*, *A. mangium*, *A. longifolia*, and *A. senegal*) grown under salt stress (Marcar et al., 1991; Hardikar and Pandey, 2008; Morais et al., 2012). Sunarpi et al. (2005) suggested that high-salt concentrations induce uptake and transport of Na^+^ and decrease K^+^ content in shoots. In vacuoles, K^+^ play pivotal role in turgor generation; however during salinity there occurs an efflux of K^+^ and influx of Na^+^ (Pottosin and Maňiz, 2002; Adams and Shin, 2014). Notwithstanding, Na^+^ has been found to help in turgor maintenance (Kronzucker et al., 2013).

Earlier studies have demonstrated that halophytes have constitutive tonoplast antiporters that facilitate Na^+^ accumulation in vacuole via SOS regulation system (Blumwald, 2000). In molecular biological studies, NHXs, vacuolar Na^+^/H^+^ antiporters to drive the Na^+^ into vacuole of halophytic species, *Aeluropus littoralis* (Zhang et al., 2008) and *Thellungiella halophila* (Vera-Estrella et al., 2005) have been well established. NHXs derived from halophyte species have been characterized, cloned, and transformed into tobacco (Jha et al., 2011), *Arabidopsis* (Li et al., 2007) and rice crop (Verma et al., 2007). We found an increase in Ca^2+^ in phyllode of *A. ampliceps* grown under salt stress, and it was in sharp contrast to reduced Ca^2+^ in other *Acacia* species when plants were subjected to salt stress (Rehman et al., 2000; Hardikar and Pandey, 2008; Patel et al., 2010). Shirazi et al. (2001) reported that Ca^2+^ accumulation peaked at EC_*e*_ = 40 dS m^–1^ in summer season, and it was 1.47 fold greater than that in plant under low EC_*e*_. Ca^2+^ alleviates salinity, protects plants and confers tolerance to salinity in glycophytes (Greenway and Munns, 1980; Hadi and Karimi, 2012). Exogenous Ca^2+^ has been reported to mitigate salinity in halophytes (Grigore et al., 2012). Ca^2+^ plays a key role in stabilization of cell wall structure, maintenance of structural and functional integrity of cell membrane, ion transport regulation, and ion-exchange behavior, besides acting as a messenger in stress signaling (Hasegawa et al., 2000; Hadi and Karimi, 2012). We observed Ca^2+^ and Na^+^ enrichment under extreme salinity condition. The increased Ca^2+^ accumulation despite Na^+^ enrichment was in sharp contrast that Na^+^ interferes with Ca^2+^ uptake under salt stress (Rehman et al., 2000). Ca^2+^ accumulation under extreme salt condition might protect *A. ampliceps* from the toxic effects of Na^+^ by activating SOS pathways, which protect against salinity-induced injury to cell membranes. But how it occurs in *A. ampliceps* is still unknown.

Free proline and total soluble sugar in phyllodes of *A. ampliceps* grown under salt stress was enriched proportional to salt concentration in the soil. It suggests a positive role of proline in osmotic adjustment under salt stress. These findings are corroborated by similar observation made earlier in leaf and root tissues of *A. ampliceps*, *A*. *mangium*, and *A. auriculiformis* grown under salt stress in a greenhouse, depending on the degree of NaCl or EC_*e*_ strength (Diouf et al., 2005; Patel et al., 2010). Halophytes accumulate more proline than glycophytes, and it has been related to the suppression of proline catabolism by proline-oxidizing enzyme, PDH (proline dehydrogenase), and enhanced synthesis of proline via pyrroline-5-carboxylate synthetase (P5CS; Taji et al., 2004; Kant et al., 2006).

Previously, studies have demonstrated that total soluble sugar, i.e., sucrose, glucose, and fructose, content was enriched in Nipa palm exposed to 16.6 dS m^–1^ EC_*e*_ as NaCl stress (Theerawitaya et al., 2014) and in the cotyledons of quinoa halophytes exposed to 200 mM NaCl for 4 days (Rosa et al., 2009a). Sucrose, the major carbon source in growing seedlings, along with glucose acts as osmoprotectant and helps in maintaining homoeostasis and biomembrane integrity during abiotic stresses (Rosa et al., 2009b). In our study, the sugar content positively correlated with Na^+^ levels in phyllodes, thereby suggesting a role in osmotic adjustment at the cellular level in salt defense mechanism as osmoprotectant (Ghosh et al., 2001). In plants, sugars do not only function as substrates for energy and carbon metabolism, but also act as osmolytes and messengers in signal transduction, and modulate growth, development, and gene expression (Rolland et al., 2002).

Na^+^ compartmentation in the vacuole organelles of mangrove species, *Bruguiera sexangula* has been proofed by ion accumulation, relating to increase in vacuole size and to regulate the activities of Na^+^/H^+^ exchanger and H^+^-ATPase (Kura-Hotta et al., 2001; Mimura et al., 2003). In the case of mild salt stress, Na^+^ compartmentation in *A. ampliceps* may play a key role as the salt defense mechanism to reduce ion toxic symptoms. Therefore, ion toxicity was found in the older phyllode, i.e., chlorosis of *A. ampliceps* when subjected to 600 mM NaCl for 9 days. In mangrove *Bruguiera parviflora*, chloroplast ultrastructure in plant grown under 400 mM NaCl was degraded as well as, pigment protein- and thylakoid protein-complexes in both content and activity were reduced, depending on the degree of salt treatment, (Parida et al., 2003) consequently to a decline in the net photosynthetic rate (Li et al., 2008). In the present study, chlorophyll pigment, chlorophyll fluorescence, and net photosynthetic rate of *A. ampliceps* were enhanced under the mild salt stress (200 mM NaCl) and then declined when subjected to severe salt stress (≥400 NaCl). Chl_*a*_ and Chl_*b*_ pigments in salt-stressed *A. auriculiformis* were degraded in relation to the level of salinity (ranging from 1.2 to 10 dS m^–1^). Parallel to our findings, F_*v*_/F_*m*_, Φ_*PSII*_, P_*n*_, g_*s*_, and E in Nipa palm (a halophytic species) were promoted by a low salt concentration (EC_*e*_ = 8.9 dS m^–1^) and then significantly dropped, especially at the high concentration (EC_*e*_ = 57.2 dS m^–1^) (Theerawitaya et al., 2014).

## Conclusion

Na^+^ and Ca^2+^ in the phyllodes of *A. ampliceps* under extreme salt stress (600 mM NaCl) were enriched, while K^+^ declined, consequently resulting in increased Na:K ratio. Na^+^ enrichment in phyllode tissues of salt-stressed plants may get stored in vacuolar organelles as Na^+^ compartmentation. Alternatively, free proline and soluble sugar were enriched in cellular levels of leaf tissues to play a key role in osmoregulation of salt defense mechanism to protect *A. ampliceps* from salt-induced toxicity. Therefore, chlorophyll degradation, chlorophyll fluorescence diminishing, and net photosynthetic rate reduction in *A. ampliceps* under severe salt stress were evidently observed, thereby leading to overall growth inhibition.

### Conflict of Interest Statement

The authors declare that the research was conducted in the absence of any commercial or financial relationships that could be construed as a potential conflict of interest.
